# Use of Molecular Genetic Analyses in Danish Routine Newborn Screening

**DOI:** 10.3390/ijns7030050

**Published:** 2021-07-26

**Authors:** Allan Meldgaard Lund, Flemming Wibrand, Kristin Skogstrand, Marie Bækvad-Hansen, Niels Gregersen, Brage Storstein Andresen, David M. Hougaard, Morten Dunø, Rikke Katrine Jentoft Olsen

**Affiliations:** 1 Center for Inherited Metabolic Disorders, Departments of Clinical Genetics and Pediatrics, Copenhagen University Hospital, 2100 Copenhagen, Denmark; 2 Department of Clinical Medicine, Faculty of Health and Medical Sciences, University of Copenhagen, 2100 Copenhagen, Denmark; 3 Metabolic Laboratory, Department of Clinical Genetics, Copenhagen University Hospital, 2100 Copenhagen, Denmark; flemming.wibrand@regionh.dk; 4 Center for Neonatal Screening, Department for Congenital Disorders, Statens Serum Institute, 2300 Copenhagen, Denmark; KSK@ssi.dk (K.S.); MABH@ssi.dk (M.B.-H.); DH@ssi.dk (D.M.H.); 5 Research Unit for Molecular Medicine, Department of Clinical Medicine, Aarhus University and Aarhus University Hospital, 8200 Aarhus, Denmark; nig@clin.au.dk (N.G.); rikke.olsen@clin.au.dk (R.K.J.O.); 6 Department of Biochemistry and Molecular Biology, University of Southern Denmark, 5230 Odense, Denmark; bragea@bmb.sdu.dk; 7 Molecular Genetics Laboratory, Department of Clinical Genetics, Copenhagen University Hospital, 2100 Copenhagen, Denmark; morten.dunoe@regionh.dk

**Keywords:** next generation sequencing, newborn screening, neonatal screening, first-tier test, second-tier test, tandem mass spectrometry

## Abstract

Historically, the analyses used for newborn screening (NBS) were biochemical, but increasingly, molecular genetic analyses are being introduced in the workflow. We describe the application of molecular genetic analyses in the Danish NBS programme and show that second-tier molecular genetic testing is useful to reduce the false positive rate while simultaneously providing information about the precise molecular genetic variant and thus informing therapeutic strategy and easing providing information to parents. When molecular genetic analyses are applied as second-tier testing, valuable functional data from biochemical methods are available and in our view, such targeted NGS technology should be implemented when possible in the NBS workflow. First-tier NGS technology may be a promising future possibility for disorders without a reliable biomarker and as a general approach to increase the adaptability of NBS for a broader range of genetic diseases, which is important in the current landscape of quickly evolving new therapeutic possibilities. However, studies on feasibility, sensitivity, and specificity are needed as well as more insight into what views the general population has towards using genetic analyses in NBS. This may be sensitive to some and could have potentially negative consequences for the NBS programme.

## 1. Introduction

**Figure 1:**
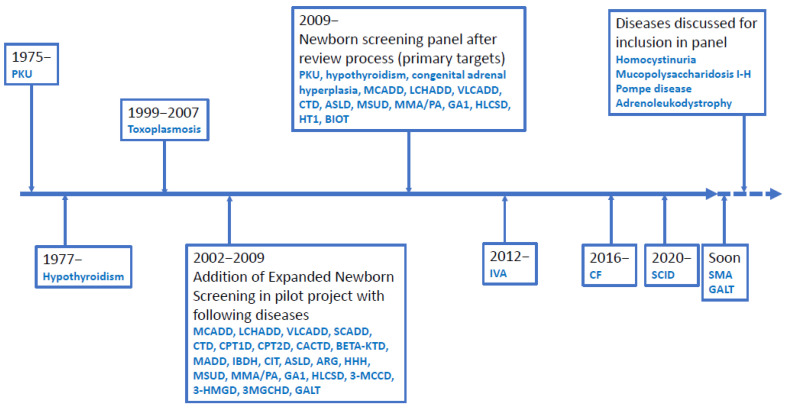
Timeline for Danish Newborn Screening. Disease abbreviations are shown in the list of abbreviations.

**Figure 2:**
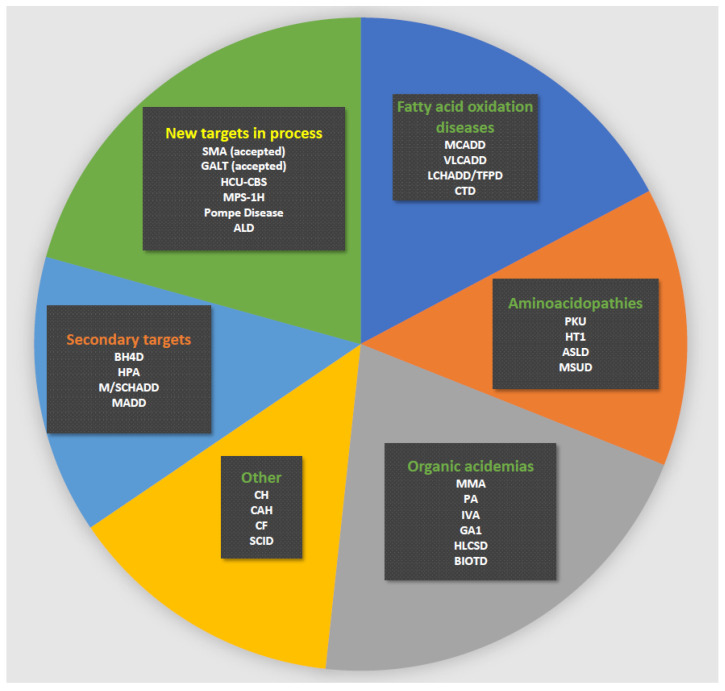
Current Danish newborn screening panel. Green heading: primary targets; orange heading: secondary targets; yellow heading: new targets in process for possible inclusion. Disease abbreviations are shown in the list of abbreviations.

Newborn screening (NBS) is a public health programme for early diagnosis of treatable, mostly genetic, diseases, such as congenital adrenal hyperplasia and selected inherited metabolic diseases (IEM) such as phenylketonuria (PKU) and medium-chain acyl-CoA dehydrogenase deficiency (MCADD). Early treatment in a latent stage of disease can, in many cases, prevent disease manifestations, which for diseases on many NBS panels includes irreversible brain and organ damage and death [1,2,3]. Internationally, NBS was started >50 years ago and in Denmark for PKU in 1975 (Figure 1). NBS for PKU became the paradigm for a well-functioning, cost-effective screening. It enabled the expansion of the disorders screened for: in Denmark, hypothyroidism was added in 1977 and expanded newborn screening (eNBS) with addition of 23 diseases tested by tandem mass spectrometry (Figure 1) was implemented in 2002. Today the Danish routine screening panel includes 18 treatable diseases (Figure 2); see also Figure 1 for a timeline of Danish NBS. In the most common set-up, the methods of screening involve the biochemical determination of analytes such as amino acids and their ratios, acylcarnitines or hormones. For confirmation of the screening positive samples, further biochemical methods are commonly used together with molecular genetic studies using new samples from the child. In recent years, molecular genetic studies have been introduced as second-tier testing before contacting the family for confirmative evaluation [1,4,5,6,7]; even the use of molecular genetic studies as first-tier analyses has been introduced when screening for spinal muscular atrophy (SMA) and severe combined immunodeficiency (SCID) [4], and further use of molecular genetics as a first-tier procedure in NBS is being discussed. In Denmark, we have recently introduced molecular genetic analyses for SCID (though, strictly speaking, not a variant analysis) as first-tier screening, and SMA has also been accepted for addition to the routine screening panel with the use of first-tier molecular genetic analyses. As a second-tier eNBS procedure, we used molecular genetic analyses on the initially obtained filter paper blood spot sample for a number of diagnoses alone or in parallel with biochemical testing. Finally, molecular genetic analyses are used as a follow-up procedure on screen positives for most diagnoses (in a new blood sample). Most diseases on many screening panels are genetic; thus, the use of molecular genetic analyses is relevant, and they have been shown to be feasible to conduct on DNA extracted from filter paper blood spot samples [8,9,10]. An important goal for using molecular genetic analyses is to decrease false positive rate [11] and to stratify infants with severe versus benign disease as has been shown for isovaleric aciduria [12]. Another goal could be to increase the adaptability of NBS making it possible to include treatable diseases without a relevant or reliable biochemical biomarker [13]. Finally, there is a general wish in many countries to increase the number of diseases on newborn screening panels and molecular genetic analyses would be able to fulfil this need because the methods are generally applicable for most inherited diseases [11].

In this manuscript, we describe the experience with and set-up for the use of molecular genetic testing in primarily eNBS in Denmark as well as perspectives for future developments.

## 2. Materials and Methods

### 2.1. General Set-Up of Danish Newborn Screening

The general set-up for Danish newborn screening (in particular eNBS) has been described in three previous papers [2,14,15]. In this paper, focus will also be on eNBS with a short note on screening for more recently included diseases, including cystic fibrosis (CF) and SCID. The historical development is shown in Figure 1, and the actual panel of diseases screened for is shown in Figure 2. Routine newborn screening is state-run, free of charge, conducted in an informed dissent set-up and covers Denmark, the Faroe Islands and Greenland, corresponding to about 62,000 births per year (www.SSI.DK/nyfoedte (accessed on 3 May 2021)). Samples consist of capillary blood collected in the local hospital by heel prick, spotted on filter paper and dried. Samples were taken postpartum day 4–9 in the pilot period of eNBS (Figure 1) (the time of sampling used routinely until 2009) and at 48–72 h during routine NBS after 2009. Primary NBS analyses are conducted in a single centralised laboratory (Statens Serum Institute, SSI) [14,15] and results are available 2–6 days after sampling. Confirmatory testing is coordinated by and conducted in Centre for Inherited Metabolic Diseases, Copenhagen. Spare blood spots have been stored since 1982 in the Danish Newborn Screening Biobank at SSI, Copenhagen. All children with a true positive screening result for an IEM are managed in Centre for Inherited Metabolic Diseases, Copenhagen; those with CF in the two dedicated CF-centres; those with congenital hypothyroidism and congenital adrenal hyperplasia in local paediatric and paediatric endocrinology departments; and, finally, those with SCID in the paediatric transplantation centre at Copenhagen University Hospital. Review of new screening targets and application to the Danish National Board of Health for inclusion in the routine panel, as well as supervision of performance and quality assurance of NBS, is conducted by the Committee for Clinical Genetics and Screening, Danish Paediatric Society and the Danish Tandem MS working group. The systematic review process of Danish NBS, laying the ground for the current Danish NBS practice, is described in a report from the Danish National Board of Health, 2008 http://sundhedsstyrelsen.dk/~/media/2FEDEEE91A014D86B06EE1B901C66A73.ashx (accessed on 3 May 2021). Decision on the current panel was based on previous screening experiences, including results from the pilot period (Figure 1) and a review with scoring of the screening potential for selected diseases [14,15]. All diseases with a scoring >the 75th centile (and some from 50th–75th centile) were reviewed further and a final list of panel diseases was made, which was effective from February 2009 (Figure 1 and Table 1) [14,15].

Screening of the first half million newborns and first million newborns, respectively, was published earlier, including analytical details for first- and second-tier as well as follow-up procedures; readers are referred to these publications for such details [2,14,15]. The study was approved by the Ethics Committee (KF 01-152/98).

For this paper we included all children born from February 1st, 2002 until February 28th 2021 [16]. The period included the eNBS pilot period until February 2nd, 2009, with eNBS performed with written informed consent, followed by NBS performed in a routine NBS program with informed dissent (see above and Figure 1). Percentage of newborns screened in eNBS changed during the period from 65% at the start to 85% at the end of pilot period and 99.85% during routine screening from 2009. Over the whole period, 1,092,450 newborns were screened. In the pilot period, 82,930 (7.6%) were not screened using the expanded screening panel (because no consent was available), but were screened for the diseases present in the routine panel (Figure 1) [14,15].

We extracted molecular genetic data for all true positives, false positives and false negatives as well as unreported results (see below) for those diseases where molecular genetic analyses were conducted on the initial filter paper blood spot sample. Overall, we conducted first-tier molecular genetic analyses for SCID (though strictly speaking, TRECs are a surrogate genetic marker and not a variant analysis). In CF, biotinidase deficiency and holocarboxylase deficiency we performed molecular genetic analyses in the initially collected filter paper blood spot sample before contacting the family. For MCADD, VLCADD, LCHADD, and IVA molecular genetic analyses were also conducted on the initially collected filter paper blood spot sample but in parallel with biochemical testing in new samples. For the remaining diseases, molecular genetic analyses were performed later in the follow-up in new samples and these are not the focus of this manuscript. For additional disorders screened for during the pilot period from 2002 to 2009, but removed from the panel in 2009, molecular genetic analyses were conducted in a few cases as described in the results section.

For other analytical details, including biochemical first tier testing, we refer to our previous publications [14,15].

### 2.2. Molecular Genetic Analyses

Conventional Sanger sequencing on DNA extracted from the original filter blood spot was used to confirm a screen positive MCADD (NM000016.4, *ACADM*), VLCADD (NM_000018.3, *ACADVL*), LCHADD/TPD (NM_000182.4, *HADHA*; NM_000183.2, *HADHB*), IVA (NM_002225.3, *IVD*), MADD (NM_000126.3, *ETFA*; NM_001985.2, *ETFB*; NM_4453.3, *ETFDH*; NM_001104577.1, *SLC52A1*; NM_024531.4, *SLC52A2*; NM_033409.3, *SLC52A3*; NM_018339.5, *RFK*; NM_025207.4, *FLAD1*)), HLCSD (NM_000411.8, *HLCS*) and BTD (NM_000060.4, *BTD*). Exonic elements and a minimum of 15 bp of the flanking intronic regions were amplified by conventional PCR and subsequently sequenced on an Applied Biosystems 3500 DX Genetic Analyzer using BigDye^®^ Terminator v1.1 Cycle Sequencing kit (Applied Biosystems, Waltham, MA, USA). Sequence data were analysed using the GeneSearch v.4.4.3 and Alamut Visual v.2.10 software. When available, parental DNA samples were investigated to determine the phase of variants. Only variants interpreted to be causative of the first-tier biochemical phenotype were reported. The coding and exon flanking sequence of the *CFTR* gene (NM_000492.4) was assessed using a commercial NGS assay (Ion AmpliSeq™ CFTR Panel) and sequenced on an Ion-S5 platform, essentially according to the manufactures description (ThermoFisher, Waltham, MA, USA). Further details can be found in [17].

Prediction of disease severity was mainly based on published or in-house data of the identified genetic variants. For novel non-published variants, clinical impact was predicted from the presumed consequence on the protein function, with loss-of-function variants (premature stop-codons, insertions/deletion creating a shift in the reading frame, etc.) categorized as severe impact. Some variants, primarily missense variants, were unknown from the literature and commonly used databases and thus did not allow for a prediction of disease severity or a specific functional consequence for the protein (variants of unknown significance). Variants were only reported if the complete genotype and the biochemical data supported a convincing conclusion. Definition of mild versus severe/classical genotypes was: mild genotypes were those where at least one of two variants from the literature was known as such or which, in unrelated individuals, have been associated with a biochemical phenotype but with no or limited clinical correlate. Classical/severe genotypes were those where both variants have a well described biochemical phenotype (published or studied in-house) and a certain associated clinical correlate with classical manifestations for the given disease.

True positive results came from newborns in whom, or in whose mothers, the suspected disease was diagnosed by confirmatory testing—see details in [2,14,15]. False positive results were from newborns in whom the suspected disease was not confirmed in the child or the mother. False negative results were from children with negative results, born in the screening period and diagnosed clinically or otherwise, e.g., by family studies, with a disease in the NBS panel.

## 3. Results

Findings during molecular genetic studies in the initial filter paper blood spot sample are described in the following and are summarized for diseases included in eNBS in Table 1.

### 3.1. Medium-Chain Acyl-CoA Dehydrogenase Deficiency (OMIM ID: 201450) (MCADD)

A total of 124 children were screened positive for MCADD, with 109 true positives and 15 false positives. Four were false negatives and were all diagnosed during family studies conducted in connection with a positive newborn screening result in a younger sibling; one of these had a classical genotype, whereas two had a mild and one had an unknown genotype. Molecular genetic analyses were conducted in all children who screened positive. Eleven of the 15 children with a false positive result were heterozygotes for a known pathogenic variant and four had no likely pathogenic variant in the *ACADM* gene; all 15 children with a false positive result had a normal or only slightly abnormal acylcarnitine profile (in carriers) on confirmative testing. For the present paper, genotypes have been classified into classical, mild and uncertain as described in the method section. Among the 109 children with a true screen positive result, the common classical variant c.985A>G was found in 140 of 218 alleles (64%) with 52 being homozygous and 36 compound heterozygous. Twenty-one children did not have the c.985A>G variant, and many of these children were of non-Caucasian descent (13 patients). Eighteen children (16%) had a known “mild” genotype [2,18], mostly compound heterozygosity for the c.199T>C variant (14 children) or c.127G>A (2 children); no child was homozygous for a mild mutation. Eleven children had an uncertain genotype, though all had abnormal acylcarnitines in follow-up samples. These samples came from non-Caucasian children in five cases. In one case, a mother was diagnosed with MCADD (homozygous for c.985A>G) because of low free carnitine in her newborn child [19].

### 3.2. Very Long-Chain Acyl-CoA Dehydrogenase Deficiency (OMIM ID: 201475) (VLCADD)

A total of 25 children were screened positive for VLCADD, with six true positives and 19 false positives. None had a false negative result. Molecular genetic analyses of *ACADVL* were conducted in 24 of the children. Eleven of the 19 children with a false positive result were heterozygotes for a pathogenic variant in *ACADVL*, while no pathogenic or likely pathogenic variant was identified in the remaining children, all of whom had normal follow-up acylcarnitines (though one had slightly raised C14:1, normalizing in a second sample taken one month after). Of the six children with true positive results, three children had a genotype of unpredictable clinical consequence and the remaining three had classical genotypes [20]. Two of the children with a genotype of unpredictable clinical consequence had pathological acylcarnitines consistent with VLCADD and have developed pronounced clinical disease consistent with early onset VLCADD disease, while the third child has clearly abnormal acylcarnitines consistent with VLCADD, but no clinical signs on standard treatment.

### 3.3. Long-Chain 3-Hydroxy Acyl-CoA Dehydrogenase Deficiency (OMIM ID: 609016) (LCHADD)

A total of five children were screened positive for LCHADD, all of whom were true positive. We found no false negative cases. Molecular genetic analyses were conducted in all. Three had well-known pathogenic variants in *HADHA* all with compound heterozygosity for the common classical variant c.1528G>C, two had a splice variant on the other allele and one had a premature stop codon variant. The remaining two children were of non-Caucasian descent and both had uncertain variants in the *HADHA* gene, with one being homozygous for a missense variant and one compound heterozygous for a missense and a splice variant. All five children had conclusive levels of 3-hydroxylated long-chain acylcarnitines suggestive of LCHADD and have developed clinical signs of LCHADD and are treated as such.

### 3.4. Multiple Acyl-CoA Dehydrogenase Deficiency (OMIM ID: 231680) (MADD)

MADD is not a primary target in Danish newborn screening, but five children turned out to be positive for more than one fatty acid oxidation disorder (MCADD and VLCADD) and were investigated. Unfortunately, only two were molecular genetically analysed using the initial blood spot. One presenting with a classical infantile clinical picture of MADD died and was therefore not molecular genetically investigated. In two children, the acylcarnitine profiles were normalized in the confirmative samples and the children were healthy and also not molecular genetically investigated. The remaining two children had persistent classical MADD acylcarnitines in follow-up samples, and in one child homozygosity for a known pathogenic variant in *ETFDH* was found. In the second child, only one *ETFB* variant of unknown significance was found in exon 3, and further investigations, including investigations of *ETFB* mRNA and Whole Genome Sequencing (WGS) with variant calling filtered to show only variants relevant for IEM, so could not establish a genetic diagnosis. Moreover, the WGS data did not reveal any variants in *ETFB* intron 2 and 3 to explain the *ETFB* exon 3 skipping events that were observed in low amounts (about 10%) of *ETFB* mRNAs. Both children were riboflavin-responsive and are developing normally.

### 3.5. Carnitine Palmitoyl Transferase 1 Deficiency (OMIM ID: 600528) (CPT1D)

Screening for CPT1D was only undertaken for a short period (2002–2007). Concerning CPT1D, 48 children were screened positive and of these, 26 were homozygous for the c.1436C>T variant common in the Inuit population. The Danish newborn screening program also covers Greenland, explaining the high frequency observed in our data. The clinical consequences associated with homozygosity for the c.1436C>T variant is unclear [21], and children with this genotype were not reported. In the remaining children acylcarnitine profiles normalized and sequencing of the *CPT1* gene was not conducted; one of these children later presented clinically, arguing for the use of second-tier CPT1 sequencing as part of NBS [22].

### 3.6. Biotinidase Deficiency (OMIM ID: 253260) (BIOTD)

A total of 79 children were screened positive, with 47 true positives, 18 false positives and 14 not reported (see below). We found no false negative children. Molecular genetic analyses were performed in all except three with a false positive result and normal biotinidase activities. Screening for biotinidase deficiency started in 2009, and enzyme testing has been the first-tier analysis in the whole of the period, but the follow-up algorithm changed: from 2009 to 2018 all children with a positive initial screening test were contacted for confirmative testing (biotinidase activity determination and molecular genetic studies); after 2018 a second-tier testing on the initial filter paper blood spot sample with sequencing of the biotinidase (*BTD*) gene was introduced, and only children with two pathogenic or likely pathogenic variants were reported. This removed all false positives. Enzyme testing in a new sample was performed at admission in all children reported to have two likely pathogenic/pathogenic variants. Cut-off for treating biotinidase deficiency was 30% residual activity.

Among the 18 children with false positive results, six children had at least one allele with the common c.1330G>C variant with two children being homozygous (with normal enzyme activities above 30% residual activity). No other likely pathogenic/pathogenic *BTD* variants were found among the remaining 12 false positive children. Among the 14 non-reported children, heterozygosity for the common c.1330G>C variant was found in a single child and no *BTD* variants in the remaining children. Among the 47 true positives, 37 children had at least one allele with the common c.1330G>C variant, with one being homozygous (with biotinidase below cut-off). Three true positives (according to biotinidase activity) were seemingly only heterozygous, with two of the variants being the c.1330G>C variant. The remaining true positives were either compound heterozygous for the c.1330G>C variant and another *BTD* variant (34 children) or homozygous/compound heterozygous for other *BTD* variants (10 children).

### 3.7. Holocarboxylase Synthase Deficiency (OMIM ID: 253270) (HLCSD)

3-Hydroxyisovalerylcarnitine (C5OH) is a marker for holocarboxylase synthase deficiency (HLCSD), common in the Faroe Islands, and a good screening target. However, C5OH is also a marker for 3-Methylcrotonyl-CoA Carboxylase deficiency (OMIM IDs: 210200, 210210) (3-MCCD), which was in the initial screening panel together with HLCSD in the pilot period [14]. After review of the screening panel, 3-MCCD was removed in 2009. From 2009, we sequenced the *HLCS* gene in all newborns with a raised C5OH and only reported those with two pathogenic alleles of the gene. As an exception, caused by the increased frequency of HLCSD in the Faroe Islands, 11 Faroese children were reported and put on biotin supplementation until sequencing results were ready; 1 turned out to have HLCSD deficiency.

There were a total (in the pilot period where 3-MCCD was reported and in the period after, where 3-MCCD was not reported) of 117 children with raised C5OH. There were 19 cases caused by 3-MCCD, among whom seven were children and 12 were mothers having a child who had a raised C5OH in their screening sample; nine were false positive for 3-MCCD (in the pre-2009 samples). In addition, one each had 3-hydroxy-3-methyl-glutaryl-CoA lyase deficiency (OMIM ID: 246450) (3-HMGCLD) and 3-methylglutaconyl-CoA hydratase deficiency (OMIM ID: 250950) (3-MGCHD). Five had holocarboxylase synthase deficiency. It is important to note that 82 children were not reported according to the above algorithm, with four being heterozygous for an *HLCS* variant. One child was false negative for holocarboxylase synthase deficiency with low C5OH (0.62 µmol/L (cut-off >1.4 µmol/L)).

### 3.8. Isovaleric Acidemia (OMIM ID: 243500) (IVA)

Screening for IVA was introduced in 2012 and 10 children were positive for isovaleric acidemia (with LC-MS/MS confirmed presence of the IVD C5 isomer) with six true screen positive children. Sequencing showed two pathogenetic variants in all six with five of them being compound heterozygous for the known mild c.941C>T variant in the isovaleryl-CoA dehydrogenase (*IVD*) gene and one being homozygous. Thus, all of them could be predicted to have a mild phenotype, confirmed by a mild clinical course in all of them. The remaining four screen-positive samples were false positive; two were heterozygous, and two had no disease-associated *IVD* variants. One of the false positives died neonatally and was found to be homozygous for a pathogenic *ENNPP1* variant associated with infantile arterial calcification—this was probably not related to the raised 3-hydroxyisovalerylcarnitine in the filter paper blood spot sample and a follow-up sample was not available. The other three children had normal acylcarnitine profiles in follow-up samples.

### 3.9. Cystic Fibrosis (OMIM ID: 219700) (CF)

During the period May 2016 (start of CF screening) until February 2021, 11,654 (3.8% of screened newborns) had a raised immunoreactive trypsinogen (IRT) used for first-tier testing. In second-tier test (testing for presence of F508del), we found 50 children homozygous for F508del, 605 children were F508del heterozygous and 330 children without F508del had IRT in fail-safe range. The 330 fail-safe children together with the 605 heterozygous children were included in our third-tier testing with next generation sequencing (NGS) of the entire cystic fibrosis transmembrane conductance regulator (*CFTR*) gene and among them we found 21 who were compound heterozygous for two pathogenic variants. One child had two candidate variants in cis (revealed by segregation analysis) and was reported as a carrier. Finally, among the fail-safe children, we found two carriers with pathogenic variants and they were reported together with the F508del heterozygotes found during second-tier testing as carriers. Thus, for CF, the policy is to report carriers in contrast to all other diseases on the screening panel. For details of Danish CF newborn screening, see [17], but a few conclusions of importance for the molecular genetic studies are that: 16% of alleles found were rare variants classified as non-pathogenic in relation to CF; the variant p.R117H was found in 1.6% of samples, but the p.R117H-5T was not found (of which only the p.R117H-5T would be reported); five (7%) of the children receiving a genetic report turned out to have a cystic fibrosis screen positive inconclusive diagnosis (CFSPID) because of the finding of variants of uncertain significance and needed long-term follow-up; two children were false negative: one with no F508del variant who did not reach fail-safe level and one child who did not reach first-tier IRT level because of meconium ileus.

### 3.10. Severe Combined Immunodeficiency (OMIM ID: 300400) (SCID)

Screening for SCID has only been part of the Danish newborn screening panel since February 2020 and is the first example of molecular genetic first-tier analyses in Danish NBS, where the amount of T-cell receptor excision circles (TRECs) is tested using real-time quantitative PCR. One year after starting screening, we have not encountered any SCID cases, but have found four children with low TRECs, two of whom were premature newborns and two children had secondary diagnoses causing low TRECs.

### 3.11. q-Spinal Muscular Atrophy (OMIM ID: 253300) (SMA)

We have prepared and validated a screening program for spinal muscular atrophy based on molecular genetic assessment for the common exon 7 deletion, and are currently waiting for political approval.

### 3.12. Other Diagnoses

For the remaining diagnoses in the Danish Newborn Screening Panel, molecular genetic studies are not a part of the screening procedures on the initial filter paper blood spot sample and are only performed as part of confirmatory testing. These diseases include methylmalonic acidemia (many OMIM IDs, but the most relevant here are: 251000, 251100, 251110); propionic acidemia (OMIM ID: 606054); glutaric aciduria type 1 (OMIM ID: 231670); phenylketonuria (OMIM ID: 261660); maple syrup urine disease (MSUD) (OMIM ID: 248600); argininosuccinate lyase deficiency (OMIM ID: 207900); hepatorenal tyrosinemia (OMIM ID: 276700); carnitine transporter deficiency (OMIM ID: 600528); and congenital adrenal hyperplasia (OMIM ID: 201910).

Here, we only want to comment on MSUD: there was a total of 57 children screen positive for MSUD and of these, three had classical MSUD. Two children presented with intermittent MSUD in the reported period, and both had normal leucine and normal leucine/phenylalanine and leucine/alanine ratios in their newborn filter paper blood spot samples. We subsequently sequenced the genes associated with MSUD (*DLD, DBT, BCKDHA, BCKDHB* and *PPM1K*) in 30 of the above 54 false positive samples. No children with two pathogenic variants were found. One was a carrier.

## 4. Discussion

In this study we showed that use of molecular genetic studies in the initial filter paper blood spot sample may decrease the false positive rate, make possible the molecular genetic filtering of diseases and stratify the severity of the disease in question in the true positive newborn. The results argue for an extension of such use of molecular genetic studies as a second-tier procedure and gives some insight as to what can be expected if used as a first-tier procedure.

### 4.1. Molecular Genetic Studies May Decrease the False Positive Rate

Our focus when introducing molecular genetic investigations in NBS has been to minimise the number of children receiving a false positive result. The Danish NBS programme performs relatively well and for the eNBS programme, the false positive rates during 2002 to 2021 were at 0.034%, corresponding to one false positive for every 2900 screened newborns. While this is relatively low, it implies that since the start of eNBS in 2002, 380 families have been investigated for a disease in the eNBS panel, that the newborn did not have. Investigations may include both expensive and invasive additional testing and a discussion of the benefit–harm ratio is relevant. While some studies cannot show any consequence of this to the families, others have shown an increased number of contacts with the health care system as well as admissions, thus suggesting a vulnerable child syndrome [11,23,24,25,26,27]. Both concerning the confirmatory investigations in the newborn period and the possible further investigations and admissions later on, the price may be considerable also from an economical point of view. Thus, there is a clear incentive to reduce the number of false positives. Some examples of success in our programme include the screening for biotinidase and HLCS deficiency. For biotinidase deficiency, we had two false positives/year before and none after introduction of second-tier genetic testing. The same was true for HLCSD screening effectively reducing the false positive rate. For 11 of 15 MCADD false positives and 11 of 19 VLCADD false positives we found heterozygosity for a pathogenic variant in *ACADM* and *ACADVL*, respectively, and the remaining had no likely pathogenic/pathogenic variants. These results strongly indicated their status as false positives even without the results of confirmative acylcarnitine profiles, which were normal or slightly abnormal (in case of true carriers). Though this did not prevent the reporting of the children because molecular genetic analyses in our set-up were conducted in parallel with biochemical confirmative testing for MCADD and VLCADD, it shows the potential of second-tier molecular genetic analyses giving sufficient data for not reporting these children; and also giving the reason for the abnormal first-tier acylcarnitines in carrier children. Data from the Norwegian NBS program also give support to these conclusions [5].

### 4.2. Molecular Genetic Studies May Filter Diseases and Disease Subtypes

As first-tier analyses are mostly biochemical and not molecular genetic, it may be difficult to separate diseases which share a common biomarker. For some of these diseases, we may wish to filter away those diseases that do not fulfil requirements for being neonatally screened for. In the view of the authors and the Danish Health Authorities, 3-MCCD is one such example, because in most individuals 3-MCCD is a biochemical finding, rather than a clinical problem [28]. Thus, we wanted to filter away and not report 3-MCCD and only report HLCS deficiency. In our cohort, one non-reported patient presented clinically in a metabolic crisis with 3-MCCD for which the child was successfully treated. This is unfortunate, but it should be seen in the following context: 81 children that were not reported and thus avoided being referred for additional testing; and 19 individuals, who were found to have 3-MCCD before we started the molecular genetic testing and who are all clinically normal today—this may be viewed as an unnecessary medicalisation. This algorithm represents an indirect filtering as we do not know whether the children have 3-MCCD—only that they do not have HLCSD. A similar algorithm using more direct filtering could be used in other settings; children with the c.199T>C variant in the *ACADM* gene have a mild phenotype, and it has been discussed—also in Denmark—whether this should be reported or at least not reported in children homozygous for the variant [29]. In the screening for MCADD, 16% had a genotype predicting a mild phenotype, which may be one reason for the three times higher number of children diagnosed with MCADD in Denmark during screening than before [2,14] and a concern in relation to possible unnecessary medicalisation. The same may be true for the common mild variant (c.941C>T) in the *IVD* gene [30]. In the Danish screening algorithm for CF the low penetrant p.R117H variant is only reported if it is found on a 5T background, also representing a filtering of the screening cohort [17]. The above are examples of situations where an entire disease or a subgroup of children with a certain disease type may be molecular genetically filtered away and not reported, possibly reducing harm. However, for such strategy we need to have an in-depth knowledge about how the molecular pathology is related to clinical manifestations or the lack of these [4]. Such knowledge is not always available [7], and we need to collect more molecular genetic data into generally available variant databases together with clinical data. Second-tier molecular genetic testing may also provide the clinician with important prognostic information, where examples may be similar to those described for the mild variants in *ACADM* and *IVD* above. Additional examples could be predictions of vitamin B12-responsive cases with MMA, Kuvan-responsive cases with PKU or riboflavin-responsive cases with MADD—all very important and prognostic information to give parents as well as to direct therapy [31].

Screening for some diseases is made difficult by the existence of enzymatic pseudodeficiency. One such disease is mucopolysaccharidosis type 1 (MPS1 deficiency, Hurler syndrome), which we consider including in the NBS panel. Pseudodeficiency alleles may be disclosed by second-tier molecular genetic testing, considerably reducing the number of children reported for confirmative testing [32] (and this number may be further reduced with parallel second-tier testing of glycosaminoglycans [33]).

### 4.3. Use of Molecular Genetic Studies: Possibilities and Drawbacks

Next-generation sequencing as part of NBS is being introduced in other countries [6]. Similar to Denmark, NGS is used in Norway as part of CF NBS and as second-tier testing for metabolic diseases [5,34]. The UK has started analysing the use of NGS in NBS [10], but otherwise experience is still limited [6,7]. There are a number of disease candidates for NGS-based diagnostics, such as MCADD, VLCADD, IVA and CF mentioned above. MADD is another candidate, which, however, may also present and exemplify controversies. At present, seven genes are known to cause characteristic MADD acylcarnitines and potential riboflavin-responsive and treatable diseases, which could be tested for with NGS as a second-tier test [35]. However, more recently, genetic deficiencies of respiratory chain complexes have also been associated with MADD-like acylcarnitines [36,37]; this challenges and possibly leads to the extension of the genetic evaluation, increasing the risk of identifying variants with unknown clinical relevance or associated with late-onset and untreatable diseases, which are not a focus for NBS [7].

#### 4.3.1. Reporting of Carriers

The reporting of carriers after third-tier NGS testing for CF is performed in the Danish NBS programme [17], but not for other diseases and—in the view of the authors—the reporting of carriers is not an aim of NBS. It is clear that second-tier molecular testing will identify carriers as they are probably enriched in false positive first-tier samples, as we observed in both CF [17] and fatty oxidation defects. In our view the finding of carriers during NBS may be used as an aetiology for being false positive and a way to reduce the false positive rate, but should not be reported. In contrast to this, reporting of carrier status may be performed relevantly in the context of preconception testing (or cascade screening early in pregnancy) as is presently being discussed as a second set-up for screening for rare, untreatable diseases in Denmark [38].

#### 4.3.2. Use as First-Tier Test

NGS in the context of a first-tier setting is being discussed and may have some advantages. Most diseases on many newborn screening panels are caused by pathogenic variants in well-known genes and diagnosis using molecular genetic analyses may seem relevant and are feasible to conduct on filter paper blood spot samples [8,9,10]. As alluded to earlier, a main goal for NBS quality development is to decrease the false positive rate and with first-tier NGS, it could theoretically be possible to obtain the same reduction in false positive rates as we observed for second-tier use of molecular genetic analyses [11]. It may also lead to stratification of infants with severe versus benign disease as for isovaleric aciduria [12]. Another goal for the first-tier use of molecular genetic methods would be to increase the adaptability of NBS making it possible to diagnose treatable diseases without a reliable biochemical biomarker [13]; thus, as shown here for intermittent MSUD, non-classical variants may not be diagnosed by routine biochemical screening [39,40,41]. Additionally, there is a general wish in many countries to increase the number of diseases on NBS panels, and molecular genetic analyses would be able to fulfil this need because the methods are generally applicable for most inherited diseases [7,11]. New therapies are being developed quickly for a number of inherited diseases with many therapies being dependent on early therapeutic initiation shortly after birth; one example is the quickly advancing therapeutic advances in spinal muscular atrophy which have been answered by a quick introduction of a molecularly based newborn screening for SMA in some countries, e.g., in Belgium [42] and very soon in Denmark.

First-tier NGS could be a means to decrease false negative rate. We found 18 children with false negative results. Two (of whom one died) had intermittent MSUD, which cannot be identified by biochemical screening [40,41]; we sequenced children with leucin above cut-off but found none. Thus, the use of second-tier molecular genetic testing would not have helped. Other children with false negative results had CTD, HLCSD and MCADD. All of these false screen negative children had pathogenic variants in the relevant genes and may have been diagnosed via first-tier NGS technology, thus potentially decreasing the false negative rate. However, in a recent paper with post hoc application of targeted whole-exome sequencing (WES) as a first-tier screening procedure, sensitivity for the included 48 inborn errors was only 88% and thus not as high as tandem mass spectrometry, which, in the same study, had a sensitivity of 99% [43]. We encountered children with low biotinidase activity who seemingly were only heterozygotes for a *BTD* variant as well as one child with a MADD biochemical profile heterozygous for an *ETFB* variant and in a set-up with first-tier targeted NGS, these children would not have been reported, increasing false negative rate. This argues for a parallel use of targeted NGS technology and biochemical methods [44], though data on sensitivity and specificity in other settings are scarce. Concerning WES specificity in an untargeted set-up, the above study [43] disclosed findings in genes unrelated to the inborn error found for the child in question, decreasing specificity to 98%—much lower than with tandem mass spectrometry, arguing for a targeted set-up if used for NBS.

We would not report carriers in a possible first-tier NGS approach, removing the ability to diagnose mothers affected with diseases such as MCADD, GA1, and CTD because of an abnormal biochemical profile in their newborn. The diagnosis of mothers is not an aim of NBS, but a number of affected mothers have been reported in the Danish NBS program with reference to the treatability of the diseases, and we have seen this as an extra benefit of NBS.

#### 4.3.3. Secondary/Incidental/VUS Findings

Use of NGS in NBS has a risk of identifying secondary/incidental findings as well as variants of uncertain significance (VUS). Risks associated with such findings are: use of increased time because of difficulties in variant interpretation postponing reporting; reporting of benign variants or variants for late-onset diseases, leading to unnecessary medicalising of the child, giving unnecessary treatment and creating patients-in-waiting among other risks. These risks are particularly high in a non-targeted set-up, while it should be minimised when the analysis is targeted exclusively to genes in the NBS panel [10,44], most of which are well-studied genes and well-represented in variant databases used for scoring the variants. However, we found a number of VUS in such known genes, including *ACADM*, *ACADVL* and *CFTR*, and this has also been shown previously for other disease genes included in NBS panels, including *PAH* gene for phenylketonuria and *GALT* for galactosemia [45]. The increasing ethnical variation in Western populations will increase the number of VUS even further, which will challenge correct interpretation in an even highly targeted NGS approach as experienced during Danish CF NBS [17]. For all 11 MCADD VUS, and for those reported for VLCADD, it would have proven difficult to report the findings if no biochemical data were available, thus increasing the false negative rate in a first-tier NGS approach. Although variant databases are available with data on specific variants, variants may still be scored very differently by different laboratories with low concordance [5,34,46,47]. All of these problems are well-known when investigating patients with a phenotype; however, during NBS, no clinical phenotype is available and a biochemical phenotype is only available if biochemical testing is conducted alongside NGS testing—with the present knowledge, this would probably be valuable to continue with [48].

#### 4.3.4. Turnaround Time

Turnaround time is a critical concern for NBS. We know from diagnostic WGS for critically ill infants that time to a result varies from 2 weeks to less than 24 h [49,50]; experiences from this are based on sequencing performed mostly untargeted, and a targeted approach for exclusively the diseases in the NBS panel could probably make analysis and evaluation more efficient [43]. If performed in parallel with tandem mass spectrometry, algorithms could be made to report immediately clearly abnormal biochemical findings such as raised succinylacetone for tyrosinemia, thus not increasing turnaround time. Using umbilical cord blood could potentially decrease the time to reporting of a positive newborn, but biochemical testing may not have sufficient sensitivity [51]. If, however, NGS technology becomes a reliable first-tier procedure, this analysis could be used for testing umbilical cord blood thus potentially reducing diagnostic age.

## 5. Conclusions and Perspectives

The use of molecular genetic technologies as part of second-tier testing is a useful tool to reduce the false positive rate while at the same time giving information about the precise molecular genetic defect to the clinician and thus informing therapeutic strategy and easing giving information about the disease and its prognosis to parents. It also gives the possibility to filter away “unwanted” diagnoses sharing a biomarker with a “wanted” diagnosis. When used in such a set-up, the valuable functional data obtained via tandem mass spectrometry or other biochemical methods will still be available and usability could be extended further via the inclusion of metabolomics or the Collaborative Laboratory Integrated Reports (CLIR) tool to further define phenotype [5,52,53,54,55]. In our view, targeted NGS technology as a second-tier technology should be implemented when possible in the NBS workflow.

First-tier NGS technology may be a promising future possibility, especially for disorders without a reliable biomarker. Pilot studies focusing on feasibility, sensitivity, and specificity should be implemented. Additionally, studies to show ways to integrate NGS technology with classical methods, metabolomics, the CLIR software or transcriptomics should be performed. Finally, we need to explore the views on the use of NGS technology as part of NBS for treatable diseases carried by the general population and in particular by those of reproductive age [27]. To conduct genetic analyses in NSB may be sensitive to some people, and it is of great importance to address all concerns, including parental fears of the inappropriate use of genetic data, in order to prevent potentially negative consequences for the NBS programme as a whole, including maintaining the high degree of trust and the more than 99.9% participation rate in the Danish NBS programme.

## Figures and Tables

**Table 1:** Number of children with screen positive, true positive, false positive, false negative and not reported results during eNBS. Classic (C), mild (M), variants of uncertain significance (VUS, V) and heterozygous variants (H) represent the genotypes found during sequencing of the initial filter paper blood spot sample (see also Methods). * This is the clinical phenotype as sequencing was not performed.

	Screen Positive	True Positive Classic (C) Mild (M) VUS (V)	False Positive Heterozygous (H)	False Negative	Not Reported
**MCADD**	124	109 80 C 18 M 11 V	15 11 H	4	NA
**VLCADD**	25	6 3 C 3 V	19 11 H	0	NA
**LCHADD**	5	5 3 C 2 V	0	0	NA
**MADD**	5	3 2 C * 1 V	2	NA	NA
**CPT1D**	48	27 1 C 26 V	21	NA	NA
**IVA**	10	6 6 M	4 2 H	0	NA
**MSUD**	57	3 3 C	54	2 (intermittent)	NA
**BIOTD**	79	47	18 pre-2018 0 post-2018	0	14 post-2018
**Raised C5OH**	117	5 HLCSD 21 other diagnoses	9	1	82 post-2009

## Abbreviations

3-HMGD	3-hydroxy-3-methyl-glutaryl-CoA lyase deficiency (OMIM ID: 246450)
3-MCCD	3-methylcrotonyl-CoA-carboxylase deficiency (OMIM IDs: 210200, 210210)
3-MGCHD	3-methylglutaconyl-CoA hydratase deficiency (OMIM ID: 250950)
ALD	adrenoleucodystrophy (OMIM ID: 300100)
ARG	hyperargininemia due to arginase deficiency (OMIM ID: 207800)
ASLD	argininosuccinate lyase deficiency (OMIM ID: 207900)
BETA-KTD	beta-ketothiolase deficiency (OMIM ID: 203750)
BH4D	biopterin cofactor deficiencies (OMIM IDs 233910, 261530, 261640)
BIOTD	biotinidase deficiency (OMIM ID: 253260)
CACTD	carnitine/acylcarnitine translocase deficiency (OMIM ID: 212138)
CAH	congenital adrenal hyperplasia (OMIM ID: 201910
CF	cystic fibrosis (OMIM ID: 219700)
CFTR	cystic fibrosis transmembrane conductance regulator
CH	congenital hypothyroidism
CIT	citrullinemia type 1 due to arginosuccinate synthase deficiency (OMIM ID: 215700)
CPT1D	carnitine palmitoyl transferase 1 deficiency (OMIM ID: 600528)
CPT2D	carnitine palmitoyl transferase 2 deficiency (OMIM IDs: 255110, 600649, 608836)
CTD	carnitine transporter deficiency (OMIM ID: 600528)
eNBS	expanded newborn screening
FAOD	fatty acid oxidation disease
GA1	glutaric aciduria type 1 due to glutaryl-CoA dehydrogenase deficiency (OMIM ID: 231670)
GALT	galactosemia due to galactose 1-phosphate uridyl transferase deficiency (OMIM ID: 230400)
IEM	inborn error of metabolism
HCU-CBS	homocystinuria due to cystathionine-beta-synthase deficiency (OMIM ID: 236200)
HHH	hyperornithinemia, hyperammonemia, homocitrullinuria due to ornithine translocase deficiency (OMIM ID: 238970)
HLCSD	holocarboxylase synthase deficiency (OMIM ID: 253270)
HPA	non-PKU mild hyperphenylalaninemia (OMIM ID: 261660)
HT1	hepatorenal tyrosinemia (type 1) due to fumarylacetoacetase deficiency (OMIM ID: 276700)
IVA	isovaleric acidemia due to isovaleryl-CoA dehydrogenase deficiency (OMIM ID: 243500)
LCHADD	long-chain 3-hydroxy acyl-CoA dehydrogenase deficiency (OMIM ID 609016)
MADD	multiple acyl-CoA dehydrogenase deficiency (OMIM ID: 231680)
MCADD	medium-chain acyl-CoA dehydrogenase deficiency (OMIM ID: 201450)
MMA	methylmalonic aciduria (many OMIM IDs, most relevant here are: 251000, 251100, 251110);
MPS-1H	mucopolysaccharidosis type 1H (OMIM ID: 607014)
MS/MS	tandem mass spectrometry
M/SCHADD	medium/-short-chain L-3-hydroxy acyl-CoA dehydrogenase deficiency (OMIM ID: 231530)
MSUD	classic maple syrup urine disease due to branched-chain alfa-keto acid dehydrogenase deficiency (OMIM ID: 248600)
NBS	newborn screening
NGS	next generation sequencing
PA	propionic acidemia due to propionyl-CoA carboxylase deficiency (OMIM ID: 606054)
PKU	classical phenylketonuria due to phenylalanine hydroxylase deficiency (OMIM ID: 261660)
PPV	positive predictive value
SCADD	short-chain acyl-CoA dehydrogenase deficiency (OMIM ID: 201470)
SCID	severe combined immunodeficiency
SSI	Statens Serum Institute
TPD	trifunctional protein deficiency (OMIM ID: 609015);
VLCADD	very long-chain acyl-CoA dehydrogenase deficiency (OMIM ID: 201475)
WGS	whole genome sequencing
